# Antimicrobial Resistance and Reduced Susceptibility in *Clostridium difficile*: Potential Consequences for Induction, Treatment, and Recurrence of *C. difficile* Infection

**DOI:** 10.3390/antibiotics4030267

**Published:** 2015-07-10

**Authors:** Simon D. Baines, Mark H. Wilcox

**Affiliations:** 1 Department of Biological and Environmental Sciences, School of Life and Medical Sciences, University of Hertfordshire, Hatfield AL10 9AB, UK; 2 Leeds Institute of Biomedical and Clinical Sciences, Faculty of Medicine and Health, University of Leeds, Leeds LS2 9JT, UK; E-Mail: mark.wilcox@nhs.net; 3 Department of Microbiology, Leeds Teaching Hospitals NHS Trust, The General Infirmary, Leeds LS1 3EX, UK

**Keywords:** *Clostridium difficile*, antimicrobial agents, resistance, reduced susceptibility, recurrence

## Abstract

*Clostridium difficile* infection (CDI) remains a substantial burden on healthcare systems and is likely to remain so given our reliance on antimicrobial therapies to treat bacterial infections, especially in an aging population in whom multiple co-morbidities are common. Antimicrobial agents are a key component in the aetiology of CDI, both in the establishment of the infection and also in its treatment. The purpose of this review is to summarise the role of antimicrobial agents in primary and recurrent CDI; assessing why certain antimicrobial classes may predispose to the induction of CDI according to a balance between antimicrobial activity against the gut microflora and *C. difficile*. Considering these aspects of CDI is important in both the prevention of the infection and in the development of new antimicrobial treatments.

## 1. Introduction

**Figure 1:**
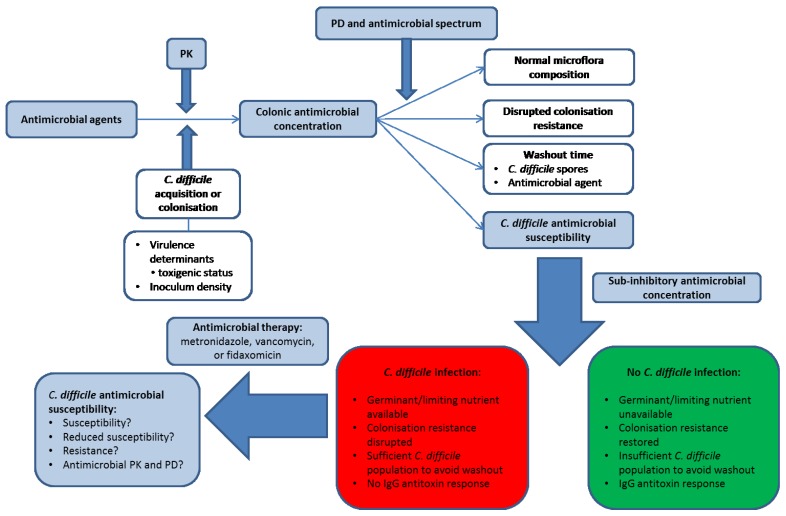
Factors involved in the development of *Clostridium difficile* infection (CDI). Blue shaded boxes involve antimicrobial agents. PK, pharmacokinetics; PD, pharmacodynamics. Antimicrobial agents differ in their pharmacokinetics (PK) and therefore in the luminal concentration that is present (and bioactive) in the human colon. In order for CDI to develop, a patient must either acquire toxigenic *C. difficile* from an exogenous source or be colonised with an endogenous strain. The antimicrobial agent disrupts the indigenous microflora to an extent governed by its antimicrobial spectrum of activity and pharmacodynamic (PD) profile, and a steady state concentration of antimicrobial agent may be reached by the end of dosing. The antimicrobial agent (and *C. difficile* spores) may then be washed out of the colon to a sub-inhibitory concentration, the speed of which depends on the steady-state drug concentration in the colonic lumen and the colonic transit time of the patient. The observation and timing of CDI depends on host immunological factors, *C. difficile*-specific factors (antimicrobial susceptibility to the inciting agent and the inoculum density of spores), and the rate and extent of recovery in the indigenous gut microflora. *C. difficile* spores do not outgrow in the presence of supra-inhibitory concentrations of antimicrobial agents, but once concentrations are sub-inhibitory then CDI may develop assuming the nutritional environment is conducive for spore germination and outgrowth (this may indicate prolonged disruption of colonisation resistance) and also that there is no IgG antitoxin response in the patient. Antimicrobial therapy for CDI may then be initiated by clinicians, the initial success of which depends on the PK/PD profile of the therapeutic agent and the susceptibility of the *C. difficile* strain. Whether recurrent CDI is subsequently observed may then depend on the degree to which the CDI therapy has negatively impacted on the indigenous gut microflora, the concentration of *C. difficile* spores remaining in the colon of the patient, whether the antimicrobial agent may persist in the colon or potentially adhere to *C. difficile* spores, and whether an IgG antitoxin response is observed.

*Clostridium difficile* infection (CDI) remains a substantial worldwide burden on healthcare systems, despite significant research and investment over the past four decades. CDI normally, but not exclusively, emerges following one or more course(s) of antimicrobial therapy for an unrelated condition, which perturbs microbial colonisation resistance within the host. Other risk factors that have been linked to the development of CDI include proton pump inhibitors and the use of anticancer agents. Antimicrobial therapy facilitates germination of *C. difficile* spores within the colon and production of toxin A and/or toxin B. *C. difficile* is the aetiological agent of pseudomembranous colitis (PMC) and is implicated in approximately 30% of cases of antibiotic-associated diarrhoea [1,2]. Most antimicrobial agents have been implicated in the induction of CDI, but clindamycin, third-generation cephalosporins, fluoroquinolones, and aminopenicillins are particularly noted for their propensity to induce CDI [3]. Despite improved clinical management strategies for CDI, healthcare costs for treating CDI remain high and were estimated at $1.1–3.2 billion in the USA [4,5]. CDI is a complex and multifactorial disease, involving the host immune response to *C. difficile* and its toxins, antimicrobial agents, their effect of the indigenous gut microflora and *C. difficile*, and the virulence of the infecting *C. difficile* strain (Figure 1). Antimicrobial agents are an intrinsic aspect of CDI; in both its induction and treatment. The widespread emergence of hypervirulent PCR ribotype (RT) 027 highlighted the potential importance of antimicrobial (fluoroquinolone) resistance in facilitating the epidemic spread of *C. difficile* clones [6].

This review will concentrate on antimicrobial susceptibility and resistance in *C. difficile* to the antimicrobial agents that induce and treat CDI, considering the prevalent ribotypes currently in circulation, mechanisms for reduced susceptibility and resistance, and pharmacokinetics of the antimicrobial agents. Furthermore, a prediction of antimicrobial agent washout will be estimated using mass-balance theory for a range of antimicrobials, to suggest how the timing of CDI (primary or recurrent) may be influenced by antimicrobial susceptibility. We will also explore how the susceptibility of important indigenous gut microflora groups may influence the risk of CDI.

## 2. Ribosomally Active Antimicrobial Agents

### 2.1. Clindamycin

Clindamycin was the first antimicrobial agent linked to pseudomembranous colitis in the mid-1970s by Tedesco and colleagues in a prospective study of 200 patients [7]. The authors reported 21% incidence of diarrhoea and 10% incidence of pseudomembranous colitis (PMC) following exposure to clindamycin. The authors noted that risk of PMC was 3–4 times greater following oral rather than parenteral clindamycin. Subsequently (in the late 1970s), the aetiology of CDI was determined when both *C. difficile* and its cytotoxic activity were demonstrated in faeces from PMC patients [1,2]. Clindamycin use has declined since clinical observations that this agent was a potent antimicrobial for inducing CDI. Surveillance for clindamycin resistance in *C. difficile* has identified multiple possible resistance mechanisms. Indeed, before highly discriminatory molecular typing methods were available, resistance to clindamycin was one characteristic that suggested that *C. difficile* subtypes existed within the same endemic ribotype (RT) 001 strains [8]. Antimicrobial susceptibility in *C. difficile* to clindamycin varies substantially between RTs and also according to geographical location. Freeman and colleagues recently published the results of a prospective susceptibility testing trial (ClosER) which encompassed 22 European countries and 953 *C. difficile* isolates [9]. Wide variations in RT prevalence were observed between countries and clindamycin resistance (MIC ≥ 8 mg/L) was present in 49.6% of isolates in multiple (including prevalent) RTs; intermediate resistance (MIC 4 mg/L) and susceptibility (≤2 mg/L) were observed in 12.7% and 37.6% of isolates, respectively, with a MIC range of 0.125 to >64 mg/L [9]. Clindamycin-resistant *C. difficile* have also been observed in other studies in Europe, Asia, North America (including RTs 017, 018, 001), and the Middle East [10,11,12,13,14,15,16,17,18,19,20,21,22,23]. Indeed, outbreaks of CDI attributed to clindamycin resistant *C. difficile* have been reported for RT027 [24,25] and other genetic types [26], with clindamycin exposure documented as a specific risk factor in CDI outbreaks. RT is not necessarily a predictor of resistance, perhaps with the exception of fluoroquinolone resistance in epidemic clones of RT027, given that the majority of susceptibility studies demonstrate susceptible and resistant strains that share the same RT.

A true understanding of MICs is governed by the susceptibility testing method employed. For simplicity, many testing studies use antimicrobial-gradient based methods such as the E-test (Biomerieux, Basingstoke, UK) or the M.I.C Evaluator strip (ThermoFisher Scientific, Waltham, MA, USA). A limitation of such methods is that definitive MICs often cannot be determined due to the pre-defined concentration range of the test strip; with the highest concentration for clindamycin being 256 mg/L. Indeed, even for gold-standard methods such as agar incorporation MIC testing, antimicrobial concentrations are doubling dilutions, often with 256 mg/L as the highest concentration; drug solubility often limits the testing of higher concentrations for many agents. Clindamycin resistant *C. difficile* with MICs of 16 to >256 mg/L often possess the erythromycin ribosomal methylase B, encoded by *erm*(B), which is often located on mobile genetic elements [27,28] and for which there are multiple genetic organisations but no clear ribotype association [12]. Erm(B) confers clindamycin resistance via methylation of bacterial 23S rRNA, and consequent prevention of drug binding and antimicrobial activity. However, clindamycin-resistant *erm*(B) negative, and clindamycin-susceptible *erm*(B) positive *C. difficile* have been reported [29]; thus, alternative mechanisms of resistance such as *erm*(B)-independent 23S rRNA methylation [12], efflux, or other unidentified mechanisms may be present in *C. difficile.*

### 2.2. Erythromycin

Erythromycin is a macrolide antimicrobial agent that inhibits protein synthesis in bacteria by binding the 50S ribosomal subunit and impairing the elongation cycle by preventing movement of the ribosome along the mRNA. Macrolides are not considered antimicrobial agents that have a strong association with CDI, although true risk data are difficult to obtain given that macrolides are often co-administered with other antibiotics; for example, with penicillins in the treatment of community-acquired pneumonia. *C. difficile* may be resistant to erythromycin via expression of *erm*(B) [12,30] or *erm*(FS) [31], methylation of 23S rRNA (*erm*(B)-independent) [12], mutation in 23S rDNA (C656T substitution in high-level erythromycin resistant and low-level clindamycin resistant, *erm*(B) negative *C. difficile*) [31], and a potential role for the *cme* efflux pump [32]. Similar to clindamycin, resistance to erythromycin varies within, and between RT and is characterised by MICs ≥ 8 mg/L (CLSI). In a recent study in Korea, of 1407 non-duplicated *C. difficile* isolates collected over 10 years, resistance (MICs 32 to >128) was seen in 3/4 of the most prevalent RT (001, 018, and 017), and in 80% of all *C. difficile* isolates [14]. Erythromycin resistance has also been demonstrated in hypervirulent RT027 [10,13,21] and 078 [33], and other prevalent RT such as 001 [10,21,34], and 017 [15]. There are 17 distinct genetic organisations of *erm*(B) (E1-E17) reported in *C. difficile* [12]. Erythromycin resistant *C. difficile* (*erm*(B) positive) appear to demonstrate reduced fitness *in vitro* in competition assays with susceptible strains, and in growth rate experiments [35]; whether such reduced fitness/virulence is present *in vivo* remains to be determined.

### 2.3. Tetracyclines

Tetracyclines inhibit protein synthesis by preventing the attachment of aminoacyl-tRNA to the ribosomal acceptor (A) site [36]. This family of antimicrobials is considered low-risk for induction of CDI, despite broad Gram-positive, Gram-negative, and anti-anaerobe spectra of activity. Tetracycline resistance in *C. difficile* was documented over 30 years ago and was demonstrated as transferable between *C. difficile* strains [37,38] and also other bacterial species [39]. Resistance to tetracyclines also varies widely between countries and with RT. In an ESGCD study in 2007, Barbut and colleagues observed no tetracycline resistant *C. difficile* in isolates from the UK and the Netherlands, 14.3% resistance in isolates from Poland, 21.4% resistance in isolates from Hungary, and 38.9% resistance in isolates from Greece; compared to 9.2% across the entire study [40]. In general, most susceptibility studies demonstrate resistance in <10% of isolates. The current CLSI breakpoint for resistance to tetracycline is ≥16 mg/L and obtaining definitive MICs is dependent on whether E-test or equivalent tests are used (maximum concentration 32 mg/L) or agar/broth MIC testing using doubling dilution concentrations. Tetracycline intermediate resistance (MIC 8 mg/L) has been demonstrated in RTs 014, 017, 078 [33,40], resistance (MIC ≥ 16 mg/L) in RTs 012 and 048 [12], and higher-level resistance (MICs 32 to >256 mg/L) reported in 32 isolates of RT012 from Sweden [41] and in an additional comparative study of isolates from Shanghai and Sweden (MICs up to 64 mg/L) [15]. Resistance to tetracyclines may be mediated by efflux proteins, ribosomal protection mechanisms, or via enzymatic inactivation of the antimicrobial [36]. In *C. difficile* tetracycline resistance most often manifests as a consequence of TetM production but may also be due to TetW [42,43]. Both TetM and TetW are cytoplasmic proteins with homology to elongation factors (EF-Tu and EF-G) that protect ribosomes from the action of tetracyclines by reducing their susceptibility to the antimicrobial [36]. The *tet* genes in *C. difficile* are carried on transposons related to Tn916 [44,45], *i.e.*, Tn5397 [44,46], Tn6190 [47], Tn6235 [48]. Spigaglia and colleagues demonstrated the concurrent presence of *tetM* and *tetW* in three isolates of RT048 and one RT012 [12], yet it is unclear if expression of both *tet* elements elicits any further reduction in tetracycline MIC. Using a MIC breakpoint of ≥8 mg/L to examine human and pig isolates from the Netherlands and UK, 75/102 human strains and 15/56 porcine strains were resistant to tetracycline [49]. All tetracycline-resistant strains contained the Tn*916*-like transposon harbouring the *tet*(M) gene. DNA fingerprinting of strains demonstrated relatedness of porcine and human strains, which is notable given the widespread use of tetracyclines in animal husbandry [49].

### 2.4. Linezolid

Linezolid is an oxazolidinone that is active against Gram-positive bacteria by inhibition of protein synthesis via targeting of bacterial 23S rRNA [50]. Linezolid is not currently used to treat CDI, but can inhibit exotoxin production in Gram positive cocci [51,52], has good activity against *C. difficile* including strains with reduced susceptibility to metronidazole [53,54,55], and inhibits cytotoxin production in complex *in vitro* models of CDI [53]. Although most *C. difficile* strains are inhibited by linezolid at concentrations far below the CLSI breakpoint for resistance (>4 mg/L), sporadic isolates with MICs of 8–16 mg/L have been reported [53,56,57]. Baines and colleagues observed two *C. difficile* (RT023 and RT067) with MICs of 8 mg/L in a study of 118 *C. difficile* isolates; further testing of 27 RT023 isolates yielded no further resistant isolates [53]. Further analysis of the two linezolid resistant isolates by PCR and sequencing did not demonstrate mutations in 23S rRNA (unpublished data). A recent study of 891 clinical isolates of toxigenic *C. difficile* yielded 9 isolates with elevated MICs (6–16 mg/L, RT001 (2/9), RT017 (6/9), and RT078 (1/9)) and PCR and sequencing identified the presence of *cfr*, which encodes a rRNA methyltransferase, in 7/9 *C. difficile* isolates on Tn6218, but not in the two RT001 isolates [56].

## 3. DNA and DNA/RNA Accessory Enzyme Inhibitors

### 3.1. Fluoroquinolones

Fluoroquinolones are synthetic derivatives of the naphthyridone molecule nalidixic acid, and were developed to increase inhibition of target molecules and broaden the antimicrobial spectrum [58]. Fluoroquinolones such as ciprofloxacin and moxifloxacin target DNA gyrase; specifically *gyrA* and *gyrB* in *C. difficile,* which encode the A and B subunits of the enzyme responsible for supercoiling bacterial DNA. Resistance to fluoroquinolones is well documented in *C. difficile* usually due to alterations in target structure via nucleotide substitutions (*gyrA* and/or *gyrB*) [6,12,59,60,61,62] within the quinolone-resistance determining region (QRDR) of DNA gyrase subunits; the role of efflux mediated resistance in *C. difficile* is unclear [12,63]. Considerable variability exists in *C. difficile* fluoroquinolone susceptibility within RT and between countries, with certain RT which are currently circulating being uniformly resistant to fluoroquinolones. The breakpoints for ciprofloxacin and moxifloxacin resistance are ≥32 mg/L (EUCAST ECOFF) and ≥8 mg/L respectively. A European prospective susceptibility testing surveillance programme (ClosER) recently reported 2011–2012 results; 40% of 953 *C. difficile* were resistant to moxifloxacin, in multiple RTs, especially RT027 and RT356 [9]. Higher-level resistance to moxifloxacin is usually characterised by MICs ≥32 mg/L, with lower-level resistance eliciting MICs of 8–16 mg/L; specific mutations within DNA gyrase subunits are associated with the magnitude of resistance. In a European surveillance study in 2008, Asp426-Val in *gyrB* was associated with high-level resistance to moxifloxacin [64], whereas in subsequent studies the same mutation was present in strains with only low-level resistance [14,65]; such observations suggest that other factors can influence resistance, possibly coupled with differences in gene expression. *C. difficile* strains may possess two concurrent substitutions in either gyrase gene*,* or a concurrent mutation in both *gyrA* and *gyrB* [65], however multiple concurrent mutations in both gyrase subunit genes have not been reported. Thr82-Ile is the most common *gyrA* substitution but Asp71-Glu, Pro116-Ala, Ala118-Ser, and Thr82-Ala also have been reported [12,64,65,66,67,68]. Mutations within *gyrB* may involve Asp426-Val, Asp426-Asn, Glu466-Val, Ser366-Ala, Leu444-Phe [12,14,59,64,65]. The acquisition of the Thr82-Ile *gyrA* substitution, in a clinical environment where fluoroquinolone use was substantial in the 1990–2000s [69], was a pivotal factor in the emergence of RT027 in the USA and Canada [6]. A recent whole genome sequencing and phylogenetic analysis of RT027 identified that the much highlighted changes in the PaLoc of *C. difficile* that were initially postulated as responsible for RT027 hypervirulence and transmission, were in fact present in pre- and post-epidemic RT027; yet the acquisition of fluoroquinolone resistance was the defining moment of the evolution and spread of the two lineages of this RT [6].

### 3.2. Rifamycins

The rifamycins achieve selective toxicity in bacteria by targeting bacterial DNA-dependent RNA polymerase. Rifamycins (rifampicin and rifaximin) have been used to treat CDI due to very low MICs in (susceptible) *C. difficile*; rifaximin (but not rifampicin) is poorly absorbed after oral administration [70]. Rifampicin resistance in *C. difficile* has been reported in 0–17.5% of isolates [9,11,12,15,17,71,72] at a breakpoint of ≥16 mg/L, with resistant isolates seen in 17/22 countries in a recent pan-European study [9]. High prevalence of resistant isolates was observed in Italy (particularly RT018 and RT356), the Czech Republic, Denmark, and Hungary, with resistant isolates in RTs 017, 027, 176, and 001/072 observed [9]. In a study of 80 clinical *C. difficile* isolates, O’Connor et al observed seven rpo*(B)* substitutions (14 *C. difficile* strains): Arg505-Lys, His502-Asn, -Tyr, -Arg, Ser488-Thr, Asp492-Asn, and Iso548-Met; therefore, several distinct substitutions, some at the same location, are associated with MICs >256 mg/L [71]. There is a bimodal distribution of rifampicin MICs in *C. difficile*, typically with results ≤0.016 mg/L and >256 mg/L [41]. Spigaglia *et al.* observed mutations in the β-subunit of RNA polymerase, encoded by *rpo*(B) (1 or 2 substitutions); specifically His502-Asn and Arg505-Lys (92% of resistant strains), Arg505-Lys alone (6% of resistant isolates), or His502-Asn alone (2% of isolates) [12]. Rifampicin resistance has been reported in multiple epidemic and non-epidemic RTs [12,22,72], including in multi-drug-resistant (MDR) strains. Mutations in *rpo*(B) between positions 488–548 may either disrupt the direct interaction between rifamycins and RpoB, or modify the rifamycin-binding pocket and therefore reduce the affinity of target for the antimicrobial [72,73]. Resistance has been described during treatment of CDI with rifamycins [74,75,76]. A RT056 isolate with a baseline rifaximin MIC of 0.002 mg/L became resistant (MIC ≥ 32 mg/L; His502-Tyr substitution) within 3 days of rifaximin therapy [76]. A second substitution (Pro496-Ser) was also identified in a second RT056 isolate and both strains persisted for 49 days, following which the patient was treated with fidaxomicin; the isolate reverted to being rifaximin-susceptible, and the patient remained asymptomatically colonised for >30 days [76].

### 3.3. Fidaxomicin

Fidaxomicin was the first new therapy to be licensed for the treatment of CDI in over 25 years; it is now recommended for use in initial or recurrent episodes of CDI [77]. Fidaxomicin is a macrocyclic narrow spectrum, bactericidal antimicrobial agent, which is poorly absorbed after oral administration, and targets bacterial RNA polymerase at a site distinct from rifamycins [78,79]. Fidaxomicin is very active against *C. difficile*, with MICs generally 0.02–0.25 mg/L [9]. Rare reports exist of fidaxomicin reduced susceptibility (MIC 2–4 mg/L) [80,81] or resistance (MIC 16 mg/L) [82]. However, even these higher MICs are approximately two orders of magnitude lower than the concentrations of fidaxomicin that are achieved in the gut lumen. Leeds *et al.* conducted laboratory studies exposing *C. difficile* to sub-inhibitory concentrations of fidaxomicin over 10 serial passages, with whole genome sequencing to characterise resistance mechanisms, and demonstrated a Glu-Arg nucleotide substitution at position 1073 in *rpo*(B) or mutation within CD22120 (a *marR* homolog); resulting in a fidaxomicin MIC of 4 mg/L [81].

### 3.4. Metronidazole

Metronidazole achieves its antibacterial action by directly damaging bacterial DNA following reduction of its nitro group once inside a bacterium. Several reduction systems have been highlighted as important for the activation of metronidazole, but perhaps most important is the pyruvate-ferredoxin/ flavodoxin oxidoreductase (PFOR) system; pyruvate is decarboxylated to acetyly-CoA and concurrently electrons are transferred to the nitro group of metronidazole by ferredoxin or flavodoxin [83]. Metronidazole has been a primary treatment option for CDI for over 30 years. However, reduced efficacy was reported by Musher *et al.* [84] in a prospective observational study of 207 CDI patients, in which only 50% of patients were successfully treated, and 22% continued to experience symptomatic CDI despite ≥10 days of treatment. Furthermore, 28% of patients experienced symptomatic recurrence within 90 days. Similar observations of reduced initial response to metronidazole and increased CDI recurrence have also been reported elsewhere [85].

Freeman and colleagues recently demonstrated that only 0.11% of 953 *C. difficile* were resistant to metronidazole (MIC ≥ 8 mg/L), with maximal MICs of 8 mg/L observed in a single RT106 isolate from the UK [9]. Interestingly, 20 isolates demonstrated reduced susceptibility to metronidazole (MIC 4 mg/L), of which 55% were RT027, isolated from the UK, Switzerland, Poland, Denmark, Germany, Denmark, the Czech Republic, and Hungary. Reduced susceptibility to metronidazole was reported in UK *C. difficile* RT001 (but not RT106 or RT027) in 2008 [86], but the clinical significance of this phenotype and the underlying mechanism is uncertain. RT027, RT106, and RT001/072 (and RT356) demonstrate elevated geometric mean metronidazole MICs compared with other RTs [9]. Metronidazole-resistant *C. difficile* have been reported in other studies [87,88,89,90] and resistance has been reported as heterogeneous, with slow-growing metronidazole resistant *C. difficile* within a population observed after extended incubation periods using E-test [91]. It is important to note that the choice of MIC methodology is crucial to the detection of reduced susceptibility to metronidazole; E-tests in particular under-estimate the MIC of metronidazole [86,92].

Despite reports of reduced susceptibility/resistance to metronidazole, and modest faecal concentrations of metronidazole (approximately 9 mg/L), CDI treatment failure has not been linked to antimicrobial resistance in *C. difficile* [93]. Until recently, mechanisms of metronidazole resistance in *C. difficile* have remained elusive; resistant strains have not been described with *nimA-J* [94,95], which codes for the reduction of the nitro group of nitroimidazoles into a poorly active amine group [96]. Three recent studies have provided important information regarding metronidazole resistance in *C. difficile* [97,98,99]. Lynch *et al.* used comparative whole genome sequencing to analyse a stable metronidazole-resistant RT027 strain and a clone that reverted to metronidazole-susceptible after freeze-thawing [98]. In addition to point mutations within sporulation (*spo0A*) and germination (*cspC*) loci, the authors demonstrated mutations in the ferric uptake regulator (*fur*), a mutation in a putative nitroreductase gene (in both RT027 strains), and a mutation in the corporphyrinogrn III oxidase gene (*hemN*) (Table 1). In a subsequent proteomic analysis of the same RT027 strains, there was no evidence of involvement of deficiencies in the PFOR system [97]. However, the authors did observe increased production of the ferric uptake regulator protein in the metronidazole-resistant RT027; Fur is a central regulator of iron homeostasis in bacteria. Moura and colleagues analysed a metronidazole-resistant RT010 *C. difficile* strain using a quantitative proteomic approach on mid-log-phase cultures [99]. No aberrations in PFOR, *hemN*, *fur*, or *nim* were detected, but an absence of ferritin was observed in the metronidazole-resistant isolate when exposed to metronidazole; therefore deficient iron storage was postulated. Additionally, reduced ferredoxin reduction, and consequently metronidazole activation, was postulated due to the significant decreased expression of butyryl CoA dehydrogenase during metronidazole exposure [99]. It should be noted that RT010 are generally non-toxigenic; also, transfer of metronidazole reduced susceptibility or resistance between *C. difficile* strains has not been demonstrated.

**Table 1:** Mutations potentially contributing to reduced susceptibility and/or resistance to metronidazole (MTZ) in *C. difficile.*

Gene/Protein implicated	Potential Contribution to Metronidazole Resistance	Ref.
Ferric uptake regulator (*fur*)	Point mutation could lead to altered binding of Fur to SOD therefore reduced oxidative stress in *C. difficile* in response to MTZ exposure.	[97,98]
Putative nitroreductase	Frameshift mutation could affect activation of MTZ.	[98]
Coproporphyrinogen III Oxidase (*hemN*)	Frameshift mutation could disrupt heme biosynthesis/metabolism, defective electron transport and reduced MTZ activation.	[98]
Ferritin	Absence in MTZ_R_ strain under MTZ pressure therefore deficient iron storage	[99]
Butyryl CoA dehydrogenase (Bcd)	Significant reduction under MTZ pressure, therefore possible reduced ferredoxin reduction and consequent reduction in MTZ activation.	[99]
Ferredoxin (2 proteins)	Reduced expression in MTZ_R_ and revertant strains, possible reduction in MTZ activation. Another ferredoxin protein was increased in expression in MTZ_R_ and revertant strains. Unclear significance.	[97]

MTZ_R_ = metronidazole-resistant *C. difficile*.

## 4. Cell Wall Synthesis Inhibitors

### 4.1. Vancomycin

Much like metronidazole, vancomycin has been a first-line therapy for CDI for the past 3 decades; vancomycin concentrations typically exceed 1000 mg/L in faeces after oral administration. Vancomycin retains good activity against *C. difficile*, including against strains with reduced metronidazole susceptibility and also epidemic strains that tend to have higher geometric mean metronidazole MICs. Vancomycin binds to the C-terminal dipeptide, D-alanyl-D-alanine, of the NAM-pentapeptide of peptidoglycan (PG) precursors during PG synthesis, so preventing transpeptidation (cross-linking) reactions between adjacent peptide side chains of adjacent PG strains. Acquired resistance to vancomycin is well studied in *Staphylococcus aureus* and *Enterococcus* spp. and results from expression of one of 9 *van* gene clusters, which encode ligase enzymes that modify the terminal D-ala-D-ala of PG precursors [100]. The expression of vancomycin resistance is controlled by a 2-component regulatory system (VanSR). *C. difficile* has been shown to possess a *vanG* homolog [101,102] within its genome (*vanG_Cd_*), which was recently assessed for its ability to confer vancomycin resistance. *vanG_Cd_* is inducible by vancomycin, but does not promote vancomycin resistance in *C. difficile*. When *vanG_Cd_* was cloned into a vancomycin-susceptible *Escherichia coli* strain, vancomycin resistance was conferred; therefore, it was postulated that there is some mechanism in *C. difficile* that prevents expression of vancomycin resistance [100]. Susceptibility to vancomycin was recently demonstrated in 97.8% of 953 *C. difficile* isolates (MIC ≤ 2 mg/L). Reduced susceptibility to vancomycin has begun to emerge over the past 5 years (*i.e.*, MICs ≥ 4 mg/L), but remains relatively sporadic. Recently, single isolates with intermediate-resistance (MIC 4 mg/L) have been seen from the Czech Republic, Ireland, Latvia, and Poland, and multiple isolates from Italy and Spain, resistant to vancomycin (MIC ≥ 8 mg/L), from multiple ribotypes (including RT027, RT126, RT356, and RT001/072) have been detected. No underlying mechanisms for such reduced susceptibility have been reported [9]. The authors of this study also commented that RT018 and RT356 (which are prevalent in Italy [9]) demonstrated notably higher vancomycin geometric mean MICs (2.00 mg/L and 2.28 mg/L, respectively) than other common ribotypes (0.62–0.95 mg/L). In a Swedish study, Noren and colleagues observed three RT002 isolates with vancomycin MICs 4-8 mg/L over a 2 year period in a patient who underwent long-term IV vancomycin therapy for *S. aureus* septicaemia [41].

### 4.2. Penicillins

Broad spectrum β-lactam antimicrobial agents are well recognised for their propensity to induce CDI, e.g., aminopenicillins and cephalosporins. Conversely, some β-lactams when given as combination products with β-lactamase inhibitors, e.g., piperacillin-tazobactam, are not noted for their propensity to induce CDI, and indeed have been used in preference to cephalosporins to reduce CDI rates [103,104]. *C. difficile* susceptibility to β-lactams varies depending on the antimicrobial, with general susceptibility to β-lactam-β-lactamase inhibitor combinations, resistance to cephalosporins (although small numbers may be susceptible), and intermediate resistance or susceptibility to aminopenicillins. Noren *et al.* reported penicillin V and piperacillin MIC_90_ of 8 mg/L and 32 mg/L, respectively, in *C. difficile* isolates from Sweden over a 15 year period, with some isolates demonstrating high-level resistance (>256 mg/L) [41].

## 5. Antimicrobial Susceptibility of the Indigenous Gut Microflora

From early *in vitro* experiments exploring colonisation resistance [105], and its association with antimicrobial therapy [106], it was clear that the indigenous gut microflora played a pivotal role in gut homeostasis and the prevention of CDI. A new antimicrobial agent to treat CDI should be: (1) assessed for its propensity to disrupt the indigenous gut microflora such that CDI may emerge; (2) as narrow spectrum as possible, ideally *C. difficile* specific; and (3) have a favourable pharmacokinetic/pharmacodynamic profile. Researchers have assessed *in vitro* and *in vivo* the effects of antimicrobial agents commonly linked to CDI; measured endpoints include which populations of the indigenous gut microflora are adversely affected or are promoted, whether *C. difficile* can be isolated and what is the duration of toxin production following spore germination [53,62,105,107,108,109,110,111,112,113,114,115,116,117,118,119,120,121,122,123,124]. Identifying a small number of key bacterial groups responsible for colonisation resistance to CDI in amongst over 500 bacterial species in the human colon is obviously an extremely challenging feat, but one that has been attempted for over two decades [105,125,126,127]. Recent research into selective microbial restoration therapy [128] and feacal microflora transplantation (for review see [129]) has demonstrated positive outcomes in the treatment of CDI. However, to design optimal interventions that are targeted at CDI, and also to minimise the risk of selection of *C. difficile*, further research is needed to delineate which are the critical microbial factors in disease pathogenesis. Recurrent CDI occurs in approximately 20% of CDI cases following treatment with metronidazole or vancomycin, and this remains a key goal for improving therapeutic outcomes.

### 5.1. Antimicrobial Agents Associated with CDI Induction

Antimicrobial agents most commonly associated with CDI cause a shift from an anaerobe-dominated gut microflora in favour of facultative-anaerobes. Within the indigenous gut microflora, alterations in the community structure may occur over time in response to diet, stresses, antimicrobial agent exposure, and age, but the total concentrations of microbes remain relatively constant. The changes in gut microflora community structure associated with the administration of selected antimicrobial agents are presented in Table 2. From these *in vitro* and *in vivo* studies it is clear that antimicrobial agents that are potent inducers of CDI elicit widespread reductions in the anaerobic microflora, particularly *Bacteroides* spp. and *Bifidobacterium* spp., with a concurrent increase in certain populations of facultative anaerobes either during or after antimicrobial dosing, e.g., *Enterococcus* spp. and lactose-fermenting *Enterobacteriaceae.*

**Table 2:** Changes in selected indigenous gut microflora populations following exposure to antimicrobial agents *in vitro* and *in vivo.*

		Negatively Impacted Populations		Positively Impacted Populations		Refs
	Antimicrobial Agent	Anaerobes	Facultative Anaerobes	Anaerobes	Facultative Anaerobes	
**Common inducers of CDI**	Clindamycin	Bifidobacteria, Bacteroides, Eubacteria, Clostridia	Lactobacilli	No effect	Enterobacteria, Enterococci	[53,107,110,111,112,114,117,130]
	Ciprofloxacin	Anaerobes overall, Bifidobacteria, *Bacteroides* spp., Clostridia	*E. coli* (LFE), Lactobacilli, Enterococci	No effect	Enterococci (PD)	[62,131]
	Moxifloxacin	Bifidobacteria, *Bacteroides fragilis* group, Clostridia	LFE, Enterococci	No effect	Enterococci (PD)	[62]
	Levofloxacin *	Bifidobacteria, *Bacteroides fragilis* group	LFE, Enterococci, Lactobacilli	No effect	Facultative anaerobes overall	[62]
	Co-amoxyclav	Bifidobacteria, *Bacteroides fragilis* group, Clostridia	No effect	No effect	Enterococci, LFE	[132]
**Infrequent inducers of CDI**	Piperacillin tazobactam	Bifidobacteria, Anaerobic cocci	Lactobacilli, Enterococci	No effect	Enterococci, Lactobacilli, Clostridia (PD)	[133]
	Piperacillin tazobactam	Anaerobes overall, Bifidobacteria, *Bacteroides fragilis* group,	Lactobacilli, LFE,	No Effect	Enterococci, Lactobacilli, Clostridia (PD)	[108]
	Mecillinam	Bifidobacteria	LFE	No effect	No effect	[113]
	Erythromycin	Bifidobacteria, Bacteroides, Clostridia	*E. coli*, Streptococci, Lactobacilli, Enterococci	Eubacteria	No effect	[134]

PD, post-dosing; LFE, lactose-fermenting *Enterobacteriaceae.* Underlined bacterial groups were substantially reduced (≥3-log_10_ cfu/mL). * 1/2 experiments, second experiment demonstrated a reduced antimicrobial effect.

Culture-independent techniques have supplemented the data generated from culture-based studies. In a study of the intestinal microbiota of 25 CDI patients compared with those for 50 matched controls, Manges *et al.*, used 16S rRNA microarray to show that the *Bacteroidetes* and *Firmicutes* phyla were associated with development of CDI [135]. *Lactobacillus* and *Clostridium* are two important members of the *Firmicutes* phylum. Additionally, Antharam and colleagues recently reported the results of a study of 39 CDI patients (*vs*. 36 non-CDI diarrhoea patients, and 40 controls), assessing the gut microflora composition, using culture-independent 454 16S rRNA Pyrosequencing [136]. The study reported marked decreases in microbial diversity and species richness, principally due to reduced *Firmicutes* phylum sequence reads. Furthermore, associations between the depletion of *Ruminococcaceae, Lachnospiraceae*, and butyrogenic bacteria in the gut microbiota and nosocomial diarrhoea, including CDI were noted [136]. 

Such changes in gut microflora community structure, based on viable count data, correlate well with susceptibility studies and the concentrations of antimicrobial agent that are observed in faeces following dosing (Table 2, Table 3 and Table 4). *Bifidobacterium* spp. are in general susceptible to penicillins, macrolides, glycopeptides, and fluoroquinolones, but variations between species are observed [137]. Some *Bifidobacterium* spp. possess resistance determinants such as *tetW* [138], and *erm*(X) [139] to confer resistance to tetracyclines and erythromycin/clindamycin, respectively. *Bacteroides* spp. vary in susceptibility to co-amoxiclav, with MIC_90_s ranging from 4 to >128 mg/L [140] and also vary substantially in their susceptibilities to fluoroquinolones [140,141]. Stiefel *et al.* raised an interesting hypothesis that antimicrobial-resistant gut microflora may be protective in the gut when a patient is exposed to antimicrobial agents [142]. A cephalosporinase-producing *Bacteroides thetaiotamicron* was dosed orally into mice for 3 days, prior to administration of subcutaneous ceftriaxone and oral *C. difficile* spores, and showed that prior colonisation with the *B. thetaiotamicron* strain inactivated intra-intestinal cephalosporin and also prevented overgrowth by *C. difficile* [142]. If key protective bacterial species within the human colon were resistant to antimicrobial agents that were active in the induction of CDI, this may potentially stabilise host colonisation resistance, although possible transfer of resistance determinants to pathogenic bacterial species is an obvious concern. In a novel recent study assessing antimicrobial-induced gut microflora changes in mice and resultant susceptibility to CDI development, and correlating changes in gut microflora in hospitalized patients, Buffie and colleagues suggested that *Clostridium scnidens* may be a key protective species against CDI [143].

### 5.2. Antimicrobial Agents to Treat CDI: Metronidazole, Vancomycin, and Fidaxomicin

Treatments for CDI also may adversely affect the indigenous gut microflora and therefore prolong the perturbation of the microflora; thus, putting an individual at risk of recurrent CDI either because persistent spores may germinate (CDI relapse) or if a new strain is acquired (CDI re-infection). The reduced efficacy of metronidazole may be to be due to a perturbed microflora [85,110,144,145,146] that is exacerbated by the CDI treatment drug, reduced or heterogeneous susceptibility to metronidazole in clinical *C. difficile* [86,91], and/or host factors including immunoresponsiveness to *C. difficile* and its toxins [147]. Mean metronidazole concentrations in faeces range from 0–26 mg/kg (approx. mg/L) [123,144,148,149,150] (Table 4) and levels decline as the colonic mucosal inflammation resolves [148]; therefore, only low, possibly sub-therapeutic, antibiotic concentrations may be present, leading to inconsistent treatment outcomes. Assuming a supra-MIC of metronidazole, bactericidal activity may be demonstrated against key anaerobic gut microflora groups (*Bifidobacterium* spp. and *Bacteroides* spp.) and this, combined with rapid decline of antimicrobial concentration following cessation of dosing (*i.e.*, washout), may allow *C. difficile* to compete for key nutrients [102] that are needed for growth and toxin production leading to recurrent infection. 

**Table 3:** Changes in selected indigenous gut microflora populations following exposure to CDI therapeutic antimicrobial agents *in vitro* and *in vivo.*

Antimicrobial Agent	Negatively Impacted Populations		Positively Impacted Populations		Refs
	Anaerobes	Facultative Anaerobes	Anaerobes	Facultative Anaerobes	
Vancomycin	Bifidobacteria, Bacteroides, Clostridia	Lactobacilli, Enterococci	No effect	LFE PD. Lactobacilli (PD)	[111,151]
Metronidazole^+^	Bifidobacteria, Bacteroides, Clostridia	No effect (one study *E. coli*)	No effect	LFE	[110,152]
Fidaxomicin	Bifidobacteria	Enterococci	No effect	LFE	[146]

PD, post-dosing; LFE, lactose-fermenting *Enterobacteriaceae.* Underlined bacterial groups were substantially reduced (≥3-log_10_ cfu/mL). ^+^ In studies where bioactive metronidazole was detected.

Very high vancomycin levels occur in faeces following oral administration (1345–1406 mg/L) [123,150]. In healthy volunteers this results in marked reductions in *Enterococcus* spp. and *Bacteroides* spp., and increased numbers of facultatively-anaerobic Gram-negative bacilli (*Klebsiella* spp., *Citrobacter* spp., and *Enterobacter* spp.) [151]. Previous investigations also indicated the emergence of intrinsically-resistant Gram-positive bacteria during vancomycin therapy [151,153]. Instillation of vancomycin in a human *in vitro* gut model reported similar alterations in bacterial community structure [114]. Vancomycin-susceptible Gram-positive bacterial groups (bifidobacteria and enterococci) were markedly reduced, while intrinsically-resistant Gram-positive groups such as lactobacilli and pediococci increased in number [114]. Interestingly, a marked reduction in *Bacteroides* spp. was demonstrated following vancomycin administration, in accordance with the observations of Edlund and colleagues [151]. Citron *et al.* demonstrated susceptibility to vancomycin in seventeen isolates of *B. fragilis* with MICs ranging from 16–128 mg/L; thus, very high concentrations of this anti-Gram-positive antibiotic are sufficient to adversely affect Gram-negative populations such as *Bacteroides* spp. [55].

Sears *et al.* analysed 175 faecal specimens from CDI patients who underwent a 200 mg twice-daily dose of fidaxomicin for 10 days and reported a mean (±SE) faecal concentration of 1396 mg/kg (±77.0) [154]. An active metabolite of fidaxomicin (OPT-1118) was also detected in faecal specimens and is also active against *C. difficile.* With fidaxomicin achieving such high faecal concentrations there is the potential that it may disrupt the indigenous gut microflora, even species with raised MICs. Susceptibility testing data indicated that fidaxomicin has good activity against *C. difficile*, *C. perfringens*, *C. innocuum* (MICs < 0.125 mg/L)*,* but also has activity against other Gram-positive bacteria such as *Bacillus* spp. (MIC 1 mg/L), *Enterococcus* spp. (MIC 4 mg/L), *Lactobacillus casei* (but not *L. acidophilus*) (MIC 1 mg/L), *Micrococcus luteus* (MICs ≤ 0.06 mg/L), *Bifidobacterium* spp. (MICs ≤ 0.25 mg/L), and anaerobic cocci (MICs ≤1 mg/L). Additionally, some anti-Gram-negative activity was demonstrated against *Moraxella cattharalis* (MIC ≤ 2 mg/L) and *Acinetobacter calccoaceticus* (MIC 1 mg/L) [155,156]. Conventional susceptibility data does not tell us the actual effect of fidaxomicin on the gut microflora, where indigenous microorganisms are exposed to very high antibiotic concentrations of fidaxomicin, and what the potential effect on colonisation resistance is.

Gut model and *in vivo* studies offer insights into the effects of CDI therapeutics on gut microflora [146,157]. In an *in vitro* gut model fidaxomicin is effective in treating simulated primary and recurrent CDI [146]; inhibition of *Bifidobacterium* spp. was seen in three of four gut model experiments and the microflora became dominated by lactose-fermenting *Enterobacteriaceae.* Despite these microflora changes, fidaxomicin instillation was not associated with recurrent CDI, possibly due to persistence of supra-MICs of fidaxomicin for 2–3 weeks after dosing cessation, so preventing *C. difficile* spore germination. No inhibitory activity against *Bacteroides* spp. was observed, as would be predicted from *in vitro* susceptibility testing [155,156]. Another way of delineating the potential for recurrent CDI associated with a therapeutic is to measure the antibiotic-mediated inhibition of *C. difficile* spore outgrowth [158].

## 6. Role of *C. difficile* Antimicrobial Susceptibility in CDI Induction and Treatment

Trying to assess the importance of the susceptibility of *C. difficile* to antimicrobial agents is not as simple as it might first appear, since CDI may result from the administration of antimicrobial agents to which the infecting strain is either susceptible or resistant. It is the timing of CDI that may differ; earlier infection may occur if a *C. difficile* strain is resistant to an antimicrobial agent. *In vitro* experiments with a human chemostat model of the colon clearly demonstrate *C. difficile* spore germination, outgrowth, and toxin production occur once bioactive antimicrobial concentrations decline to sub-MIC levels [110,111]. There are of course many other factors than simply antimicrobial concentrations that interplay to facilitate CDI (Figure 1).

**Figure 2:**
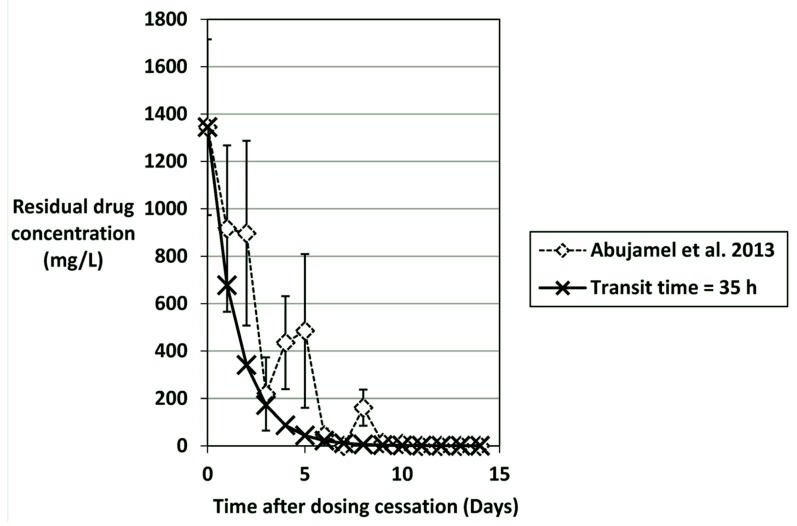
Predicted washout of vancomycin (mg/L) using a mass-balance calculation assuming a gut transit time of 35 hours (dilution rate = 0.029 h^−1^). Vancomycin concentrations (mean ± SE) are from Abujamel *et al.* [123,161] and mg/kg were assumed to equal mg/L.

By using a mass-balance analysis of faecal concentrations of antimicrobial agents, it is possible to predict how quickly antimicrobials will be washed out of the gut and therefore analyse the potential importance of elevated MICs (Table 4). It should be noted that these estimations assume no binding of or inactivation of the antimicrobial. Human colonic transit times vary from 20–120 h, and are often slower in the elderly [159,160]. Selecting an appropriate gut transit time and corresponding dilution rate (1/transit time, h^−1^) is complicated by the variability between subjects. For the present predictions, the gut transit time was based upon the rate of washout of vancomycin observed in the study of Abujamel *et al.* (vancomycin concentrations kindly supplied by Dr Curtis Donskey) [123]. By selecting a gut transit time of 35 h (Dilution rate = 0.029 h^−1^), washout to <1 mg/L vancomycin would be achieved by day 11 after dosing ceased, which reflects the data published in [123] (Figure 2). Using this same method it can be calculated that for a *C. difficile* strain with a vancomycin MIC of 4 mg/L, spores would be able potentially to germinate and outgrow 2 days before a strain that had an MIC of 1 mg/L; *i.e.*, day 9 *vs.* 11 post cessation of vancomycin dosing, assuming a faecal concentration of 1350 mg/L at the end of dosing. This may therefore provide a competitive advantage for strains with reduced vancomycin susceptibility; strains with an MIC of 8 mg/L could potentially germinate/outgrow a day earlier still.

Importantly, germination, outgrowth, and proliferation of *C. difficile* will also be part-determined by the extent of persisting colonisation resistance disruption that is either due to the original CDI inducing antibiotic(s) and/or the therapeutic agent. If we consider a “fidaxomicin-resistant” *C. difficile* strain with an MIC of 16 mg/L, spores could germinate/outgrow 7 days after cessation of fidaxomicin dosing, whereas susceptible strains (MIC 0.06 mg/L) would be inhibited from outgrowing until 15 days after cessation of fidaxomicin dosing if we assume concentrations in faeces were 1400 mg/L and no additional persistence due to spore binding or sequestration within gut biofilms. For metronidazole reduced susceptibility strains (MIC 4–8 mg/L) there is the possibility that luminal metronidazole concentrations during CDI therapy would be sub-MIC and therefore not inhibit *C. difficile.* If we assume that 15 mg/L of metronidazole was present at the end of a dosing regimen, then spores of *C. difficile* with MICs of 4 or 8 mg/L would be able to germinate/outgrow after 1 to 2 days following cessation of metronidazole dosing, compared to 5 days for a strain with an MIC of 0.5 mg/L. Abujamel *et al.* commented that there was a 3 week vulnerable period for re-establishment of *C. difficile* colonization after CDI treatment with vancomycin or metronidazole [123]. This observation is consistent with predicted concentrations of vancomycin and metronidazole falling below the MIC for *C. difficile* that are susceptible to vancomycin/metronidazole.

These same principles can be applied to the other antimicrobial agents which commonly induce CDI (Table 4), with an obvious selective advantage for *C. difficile* with reduced drug susceptibility or resistance, when proliferation may occur before gut microflora colonisation resistance has normalised.

**Table 4:** Predicted washout of antimicrobial agents from the human colon using a mass-balance calculation assuming a gut transit time of 35 h (dilution rate = 0.029 h^−1^) and representative steady-state concentrations observed during antimicrobial dosing based on published data.

Antimicrobial Agent	Representative Steady-State Concentration (mg/L)	Washout Time (days) to Achieve Residual Antimicrobial Concentration						Faecal/bile (B) Concentrations *in vivo* (mg/kg)	Refs
		32 mg/L	16 mg/L	8 mg/L	4 mg/L	2 mg/L	1 mg/L		
Metronidazole	15	NR	NR	1	2	3	4	0, 9.3, 26	[123,144,148,149,162]
Vancomycin Fidaxomicin	1350 1400	6	7	8	9	10	11	1345,1406 1396	[123,150] [154]
Clindamycin Ciprofloxacin	150	3	4	5	6	7	8	33.9(B), 97, 147.4, 203.8 136.8, 168.5, 891	[130,163,164,165] [131,165,166]
Erythromycin Moxifloxacin	500	5	6	7	8	9	10	330, 978 573.3	[134,164] [167]
Rifaximin	8000	9	10	11	12	13	14	7961	[168]

NR = Not a relevant concentration for this antimicrobial agent.

## 7. Conclusions

So what does all of this mean for existing and new CDI treatments? The preferred characteristics of the ideal CDI therapeutic are easy to state: Potent bactericidal activity against all *C. difficile* ribotypes, low potential for mutational resistance, narrow spectrum activity that results in minimal gut microflora disruption, a good pharmacokinetic profile that yields high enough luminal concentrations sufficient to inhibit/kill *C. difficile* that leads to washout of residual toxins and spores (although biofilm retention of toxins/spores may complicate this aim), and finally no recurrence of CDI after excretion of antibiotic. The efficacy of the antimicrobial against *C. difficile* and the indigenous gut microflora, and likelihood of recurrence can be assessed using *in vitro* models and to a lesser extent in animal models of CDI, but human trials are where the most pivotal data are generated.

One aspect not evaluated extensively in this review is the effect of the antimicrobial agent on the physiology of *C. difficile* and the normal gut microflora at the levels of gene transcription and translation. Antimicrobial agents have been studied for their effects against *C. difficile* at sub-MIC in several studies at the transcriptional and also the whole organism levels [169,170,171,172] and been shown to affect the toxin production and expression of colonisation structures of *C. difficile.* These studies demonstrate interesting observations but based on the faecal concentrations presented in Table 4 should be interpreted with caution as they rely on vegetative *C. difficile* being exposed to sub-MIC which may not be the case in all CDI patients. Novel antimicrobial agents, both CDI therapeutics and agents for other infections, would ideally not stimulate *C. difficile* to express virulence determinants or elevate their levels of expression. However, such considerations are not necessarily criteria for discounting the development of an agent and these types of studies are not often performed in drug development programmes. In terms of preventing CDI, it is imperative that resistance development in *C. difficile* is limited as this provides *C. difficile* with a competitive advantage as it may facilitate CDI earlier in the course of a therapeutic antimicrobial. Reduced susceptibility to metronidazole may potentially result in therapeutic failure due to low bioactive drug concentrations in the colon and could predispose to greater likelihood of recurrent CDI, although clinical data supporting this hypothesis are lacking. Conversely, reduced susceptibility to fidaxomicin and vancomycin is unlikely to affect primary treatment efficacy for CDI due to very high luminal concentrations in the gut. Antimicrobial agents are integral in the aetiology of CDI and although resistance is not a prerequisite for CDI to occur, there is now substantial evidence that mutation to a resistant phenotype may confer a significant selective advantage within the gut ecosystem. Limiting the spread of antimicrobial resistance in *C. difficile* should be a primary goal for clinicians and scientists, and prospective monitoring of resistance phenotypes and genotypes are essential in achieving this.
